# Academic Resilience Among Vocational High School Students in Collectivist Culture: The Role of Intolerance of Uncertainty and Academic Self-Efficacy

**DOI:** 10.3390/bs16040560

**Published:** 2026-04-08

**Authors:** Banu S. Ünsal Akbıyık, İhsan İlker Çitli, Melis Melek Kahveci

**Affiliations:** 1 Department of Tourism Guidance, Faculty of Tourism, Kocaeli University, 41001 İzmit, Kocaeli, Türkiye; 2 Department of Guidance and Psychological Counseling, Faculty of Education, Medipol University, 34810 Beykoz, İstanbul, Türkiye; ihsan.citli@medipol.edu.tr; 3 Department of Tourism Management, Faculty of Tourism, Kocaeli University, 41001 İzmit, Kocaeli, Türkiye; melismel2016@outlook.com

**Keywords:** intolerance of uncertainty, academic self-efficacy, academic resilience, vocational high school, collectivist culture

## Abstract

Academic anxiety frequently emerges when students perceive academic demands as uncertain, uncontrollable, or threatening. Intolerance of uncertainty is widely recognized as a key cognitive antecedent of such anxiety, influencing how learners appraise stressors and mobilize coping resources. This study investigates the relationships among intolerance of uncertainty, academic self-efficacy as a coping mechanism, and academic resilience among vocational high school students in a collectivist educational context. Data were collected from 387 vocational high school students across Istanbul, Turkey via online forms. Contrary to expectations, the results revealed that intolerance of uncertainty positively affects academic self-efficacy. Furthermore, academic self-efficacy was positively associated with academic resilience. Academic self-efficacy partially mediated the relationship between these two variables. These findings provide new insights into how uncertainty is managed in collectivist educational contexts and suggest directions for future educational practices and research.

## 1. Introduction

Uncertainty refers to unknown information about an event or situation that cannot be predicted in advance (Brashers, 2001; P. Schweizer, 2020; S. Schweizer et al., 2023). While some individuals can tolerate uncertainty, others struggle with it, exhibiting negative emotional, cognitive, and behavioral responses such as anxiety and stress (Sandhu et al., 2023). Adolescence is a developmental period characterized by high levels of uncertainty due to physical, emotional, social, and personal changes.

In this context, the extent to which adolescents can tolerate uncertainty is an important issue. Adolescents with high intolerance of uncertainty are reported to struggle with problem-solving and with coping strategies, to experience increased levels of stress and anxiety, and to suffer decreased academic performance compared with adolescents with low intolerance of uncertainty (Lei et al., 2022).

Considering the rapidly changing conditions and high levels of uncertainty in the 21st century, adolescents encounter more uncertain situations than previous generations did. An adolescent born in the mid-2000s faces economic crises, educational disruptions, social isolation caused by the COVID-19 pandemic, rising living costs, and political tensions. Additionally, the employment crisis and heightened feelings of insecurity due to developments in Syria and Ukraine further exacerbate these unfavorable conditions, particularly for adolescents living in Turkey (Sever & Tatlıcıoğlu, 2024).

This study explores how adolescents’ intolerance of uncertainty affects their academic resilience. Adolescents with high intolerance of uncertainty are less capable of coping with uncertainty due to their heightened anxiety, stress, and negative emotional, cognitive, and behavioral responses (Shihata et al., 2016; C. Y. Chen & Hong, 2010). In other words, intolerance of uncertainty disrupts functionality and reduces resilience levels (Tang, 2019). Additionally, this study investigates the mediating role of academic self-efficacy between intolerance of uncertainty and academic resilience.

Resilience is the ability to withstand risks and overcome adversity, or main sources of stress that arise from childhood to old age (Windle, 2011). A resilient person is able to adapt positively to life despite all adversities and challenges, and achieve favorable outcomes (Wu et al., 2013). A student is considered academically resilient if s/he is able to achieve high academic success despite stressful events and unfavorable life circumstances that could potentially result in academic failure (Ye et al., 2021; Freiberg-Hoffmann et al., 2024). Academic self-efficacy and academic resilience are among the most significant factors that determines students’ success (Aliyev et al., 2021; Zysbergf & Schwabsky, 2021). Academic self-efficacy is strongly linked to students’ social and psychological development and has a positive correlation with academic success (Honicke & Broadbent, 2016). High academic self-efficacy enhances the quality of a student’s strategic and analytical thinking skills and motivation, while also fostering resilience, perseverance, when faced with adverse or difficult conditions (Bandura, 2001). In the present study, academic self-efficacy is conceptualized as a coping-related personal resource that may help students manage uncertain academic demands. However, coping processes themselves were not directly examined in the structural model.

Prior research has consistently demonstrated the mediating role of academic self-efficacy in the relationship between adverse life circumstances, psychological well-being, and resilience among university students (Sabouripour et al., 2021). Moreover, a substantial body of literature has established a robust association between academic self-efficacy and academic resilience across diverse student populations (Hamill, 2003; Batur & Baki, 2022). Additionally, studies investigate the associations between intolerance of uncertainty and personality traits (Fergus & Rowatt, 2014; Yang et al., 2015; Bongelli et al., 2021), depression and anxiety disorders (Dugas et al., 2022).

To our knowledge, few studies have simultaneously examined the relationships among intolerance of uncertainty, academic self-efficacy, and academic resilience. Furthermore, the study focuses on vocational high school students, a group often underrepresented in academic research despite their educational vulnerability. These schools often serve students from lower socioeconomic backgrounds, who experience persistent disadvantages in accessing educational opportunities, while high dropout rates in vocational education and training (VET) further exacerbate their vulnerability, underscoring the need for research on academic self-efficacy and resilience in vocational contexts (Nika, 2025; Böhn & Deutscher, 2022; Schmid & Haukedal, 2022). Moreover, the limited number of studies regarding adolescents’ intolerance of uncertainty, along with the fact that adolescents differ from adults in terms of neurodevelopmental characteristics, make it difficult to generalize findings from adult-focused studies to adolescents, thereby highlighting the significance of the present study (Shihata et al., 2016; Krain et al., 2006). Although conducting this research in Turkey—a country with a collectivist culture that values shared goals, cooperation, and a sense of community—was not the study’s primary objective, it adds another dimension to its significance.

## 2. Conceptual Framework

### 2.1. Intolerance of Uncertainty

Intolerance of uncertainty refers to an individual’s tendency to interpret ambiguous or unpredictable situations as threatening and risky. This trait is typically associated with negative emotional, cognitive, and behavioral reactions in the face of uncertainty (Birrell et al., 2011).

The construct comprises two related but distinct dimensions: prospective intolerance of uncertainty and inhibitory intolerance of uncertainty (Carleton et al., 2007; Carleton et al., 2012). Prospective intolerance refers to the desire for predictability and active inforSabouration seeking aimed at reducing ambiguity. Individuals high in prospective intolerance tend to engage cognitively with uncertain situations in order to regain control. In contrast, inhibitory intolerance reflects behavioral paralysis or avoidance in the face of uncertainty, leading to inaction or withdrawal (Birrell et al., 2011). In academic contexts, particularly among adolescents frequently confronted with evaluative ambiguity, intolerance of uncertainty may manifest not only as avoidance (inhibitory dimension) but also as increased cognitive engagement and information-seeking efforts (prospective dimension). Distinguishing between these dimensions is particularly relevant in understanding how intolerance of uncertainty may differentially relate to academic life (Akkoç et al., 2025).

Intolerance of uncertainty can be described as the tendency to perceive uncertain situations as threatening (cognitive), as sources of worry or anxiety (emotional), and as situations to be avoided or rejected (behavioral) (Dugas et al., 2001; Grenier et al., 2005). Individuals with a high intolerance of uncertainty are likely to avoid uncertain situations and conditions, which can result in heightened levels of anxiety. According to Bandura’s (1997) social cognitive theory and the transactional model of stress and coping reactions (Lazarus & Folkman, 1984), two appraisal processes are discussed for evaluating a situation or event. The primary appraisal process is related to one’s perception of a situation or event as a threat (Voitsidis et al., 2021). The secondary appraisal process is defined as the evaluation of an individual’s capacity to cope with perceived threats. Intolerance of uncertainty is one of the primary appraisal process related to one’s tendency to perceive events and situations as threatening (Folkman et al., 1986). Self-efficacy emerges from the second appraisal process which involves evaluating how to cope with this threatening situation.

Adolescents with high intolerance of uncertainty tend to perceive situations as more threatening or risky than those with low intolerance of uncertainty. S/he frequently develops negative perceptions of their own coping skills. Such negative perceptions are expected to shape their beliefs regarding their academic self-efficacy (Kestler-Peleg et al., 2023; Xu & Tracey, 2015). In a study conducted among high school students in Turkey, intolerance of uncertainty was also found to negatively affect students’ academic life satisfaction (Odacı et al., 2023). A student with high intolerance of uncertainty is more likely to experience a negative impact on their academic self-efficacy when faced with stressful or anxiety-inducing academic situations, as compared to a student with low intolerance of uncertainty, because they have difficulties in evaluating coping strategies for such situations.

### 2.2. Academic Self-Efficacy

Bandura (1997) defines self-efficacy as “a person’s belief in their ability to organize and carry out the necessary actions in a specific situation”. Self-efficacy, as conceptualized in Bandura’s social cognitive theory—particularly through the principle of reciprocal determinism—suggests that behavior is shaped by the dynamic interaction between personal, environmental, and behavioral factors (Pastorelli et al., 2001; Liu et al., 2022). Personal factors encompass cognitive, emotional, or biological components, whereas environmental factors include reinforcers, feedback, the behaviors of others, and situational conditions. Behavioral factors refer to a person’s actions and choices. Self-efficacy is formed by the cognitive component, which is central to the functioning of reciprocal determinism (Bandura, 1997). It guides a person in making decisions and engaging in tasks with appropriate effort. Self-efficacy is constructed through individuals’ beliefs in their own capabilities (Aliyev et al., 2021).

Self-efficacy is a dynamic construct that may vary across tasks and conditions. Academic self-efficacy is conceptualized as a student’s perception of their confidence in achieving academic goals (Bandura, 2001). Students who exhibit low levels of academic self-efficacy are likely to perform less inclination toward learning, are unable to give adequate attention to school related responsibilities, and are either unwilling to face the challenges they encounter or make no effort to overcome or succeed in these challenges (Kristensen et al., 2023; Bandura, 1997). On the other hand, students with high academic self-efficacy, when faced with challenges, tend to study longer and experience fewer negative emotional responses.

These students accept challenging and demanding tasks, demonstrate high levels of motivation to complete them, and persist (Bandura, 2001; McLaughlin et al., 2008; Zysbergf & Schwabsky, 2021). The literature shows that academic self-efficacy is positively related to various outcomes of academic success, such as grade point average, class participation, choosing challenging courses, and persisting in difficult tasks (Mercer et al., 2011; Galyon et al., 2012; Zysbergf & Schwabsky, 2021).

As individuals’ self-efficacy increases, they are more likely to stimulate cognitive mechanisms that, under adverse conditions, enhance their analytical thinking skills, boost motivation, and foster perseverance in the face of failure (Bandura, 2001). Individuals with high self-efficacy believe in their abilities and potential, persist in achieving their goals, and act decisively to succeed. Such individuals do not internalize negative emotions about themselves or their competencies (Cassidy, 2015). They are also willing to seek help when necessary and persist through adversity, even in high-risk situations (Schwarzer & Warner, 2012) and academic self-efficacy may function as a protective cognitive resource, strengthening students’ perceived coping capacity and buffering the negative effects of threat appraisal (Sabouripour et al., 2021).

### 2.3. Academic Resilience

Resilience is conceptualized as one’s capacity to effectively overcome personal weaknesses and environmental adversities, while succeeding physically and psychologically despite all adverse conditions (Rudd et al., 2021). Resilience, in essence, refers to the inner strength, self-restorative capacity, and adaptability that allow individuals to face negative life circumstances and risk factors (Wu et al., 2013; Abiola & Udofia, 2011).

Academic resilience refers to the ability to attain academic success and good academic performance despite encountering negative experiences, challenging environmental conditions, and stress-related risk factors during one’s education (Alva, 1991; Benard, 2004; Cassidy, 2016). The literature indicates that resilience is not a unidimensional concept, and should be examined in a multidimensional context-specific manner. Academic resilience refers to one’s ability to transform negative academic experiences into positive outcomes through cognitive, emotional, and behavioral mechanisms. It is examined under three dimensions: perseverance, help-seeking, and negative affect (Cassidy, 2016). Research has shown that students with high academic resilience approach the learning process differently from those with lower academic resilience. Such students spend more time and effort on studying, as well as have higher homework completion rates (Finn & Rock, 1997; Lee et al., 1991) and arrive on time for class, actively participate in lessons, and engage more in school activities and academic events. (Finn & Rock, 1997; Borman & Overman, 2004; Romano et al., 2021; Ayala & Manzano, 2018). Academic resilience, as measured by the ARS-30, reflects a constellation of psychosocial characteristics including perseverance, adaptive help-seeking, and regulation of negative affect (Cassidy, 2016). These components represent a combination of psychosocial resources that enable students to persist, seek support, and regulate emotional responses in the face of academic adversity.

Intolerance of uncertainty may influence these components by shaping how students appraise and emotionally respond to ambiguous academic demands. Academic self-efficacy, in turn, may strengthen perseverance, promote adaptive help-seeking, and reduce negative emotional reactions by enhancing students’ belief in their coping capacity. Thus, the relationships examined in this study reflect interconnections among appraisal processes, protective beliefs, and resilience-related behavioral tendencies.

Resilience, defined as the ability to limit negative responses to stress and adapt to challenging situations (Waxman et al., 2003), is hindered by high intolerance of uncertainty. In other words, individuals with high intolerance of uncertainty tend to have low self-efficacy in overcoming adversity and may not struggle to persist in the face of difficulties. Compared to students with low intolerance of uncertainty, those with high intolerance may feel less confident in their academic abilities when facing uncertainty and avoid challenges, making it harder for them to demonstrate academic resilience.

Based on the theoretical framework outlined above, the following hypotheses were proposed:

**H1.**  *Intolerance of uncertainty negatively and significantly affects academic self-efficacy.*

**H2.**  *Academic self-efficacy positively and significantly affects academic resilience.*

**H3.**  *Academic self-efficacy serves as a mediating variable between intolerance of uncertainty and academic resilience. Increased intolerance of uncertainty reduces academic self-efficacy, leading to lower levels of academic resilience.*

## 3. Methodology

### 3.1. Data Collection and Participants

The researchers obtained ethical approval from the relevant institutional authorities prior to data collection. Since the participants were aged between 14 and 19 years, special permission was granted by the Turkish Ministry of National Education to conduct the study in vocational high schools. Following this approval, the researchers met in person with the principals of the tourism and hospitality vocational high schools who agreed to participate in the study. With the support of school administrators and teachers, the researchers distributed the survey links to students through their official school e-mail addresses. Participation in the study was voluntary and anonymous, and all participants provided informed consent before completing the survey. Socio-demographic variables included information about participants’ parents’ educational levels as indicators of family background.

Participants included 2000 students enrolled in vocational high schools focused on tourism and hospitality across four central districts of Istanbul, Türkiye (Küçükçekmece, Zeytinburnu, Beşiktaş, and Şişli). These districts were selected due to their high concentration of vocational schools specializing in tourism and hospitality education. Students were invited via school e-mail lists and social media posts distributed through school counselors and administrators. Participation was voluntary and informed consent was required. A nonprobability sampling approach combining convenience and purposive elements was used, to target students who were both easily accessible and who met the specific criterion of studying in tourism and hospitality programs. A total of 387 responses were initially received, corresponding to a response rate of approximately 19% of the target population. During the data screening process, surveys containing substantial missing data (more than 20%) were excluded from the analysis, resulting in a final analytical sample of 347 students. Of the valid respondents, 68.1% were female (*n* = 233) and 31.9% were male (*n* = 109).

Participants ranged in age from 14 to 19 years (M = 16.92, SD = 0.70). Regarding grade level, 76.0% of participants were in 12th grade, 16.7% in 11th grade, 4.1% in 9th grade, and 3.2% in 10th grade. To evaluate the adequacy of the sample size, an a priori power analysis was conducted using G*Power 3.1 for the structural paths of the proposed model. Assuming a medium effect size (f^2^ = 0.15), α = 0.05, a statistical power of 0.95, and two predictors, the analysis indicated that a minimum sample size of 107 participants was required. The final sample therefore provides sufficient statistical power for the analyses. In addition, the sample size exceeds commonly recommended thresholds for structural equation modeling, which suggest that samples above 200 are generally adequate for stable parameter estimation (Kline, 2011). Furthermore, SEM studies are often recommended to include approximately 10–20 participants per estimated parameter (Hair et al., 2014), a criterion also satisfied in the present study.

Socio-demographic data indicated that 81.3% of mothers and 76.3% of fathers had completed high school. Additionally, 12.6% of mothers and 16.7% of fathers held an associate or undergraduate degree, while 6.1% of mothers and 7.0% of fathers had completed only primary education. With respect to their perceived economic status, 79.5% of students reported moderate income levels, 17.8% reported high income levels, and 2.6% reported low income levels. Regarding siblings, 55.0% had one sibling, 32.5% had two, 9.6% had three, and 2.9% had four or more. The average GPA on a 5-point scale was 2.72 (SD = 0.57).

### 3.2. The Measuring Instruments

A structured, self-administered questionnaire was developed to measure the key variables in this study. The questionnaire consisted of four main sections: (1) intolerance of uncertainty (2) academic resilience (3) academic self-efficacy (4) demographic information

*Intolerance of uncertainty:* This construct was measured using the Intolerance of Uncertainty Scale developed by Carleton et al. (2007), and adapted into Turkish by Sarıçam et al. (2014). The scale includes 12 items rated on a 5-point Likert scale (1 = strongly disagree, 5 = strongly agree) and comprises two subdimensions: prospective intolerance of uncertainty (7 items) and inhibitory intolerance of uncertainty (5 items). Items included statements such as “Uncertainty makes me vulnerable, unhappy, or sad”, “Uncertainty stops me from having a firm opinion.” 

*Academic Resilience:* The Academic Resilience Scale developed by Cassidy (2016) and adapted into Turkish by Aliyev et al. (2021) was used to assess academic resilience. The scale comprises 30 items across three subscales: perseverance (14 items), adaptive help-seeking (9 items), and negative affect and emotional response (7 items). Items were rated on a 5-point Likert scale (1 = strongly disagree, 5 = strongly agree), with 20 items reverse-scored. Items included statements such as “I would use the situation to motivate myself”, “I would use my past successes to help motivate myself”, “I would probably get annoyed”. Higher scores indicate greater academic resilience. Reliability and validity statistics for the present study are reported in Table 1. Because the negative affect dimension of the Academic Resilience Scale demonstrated inadequate model fit in the confirmatory factor analysis (see Table 2), this dimension was excluded from the final measurement model. Therefore, academic resilience was operationalized using the perseverance and adaptive help-seeking dimensions.

*Academic Self-Efficacy:* This construct was measured using the Academic Self-Efficacy Subscale from the Child-Adolescent Self-Efficacy Scale developed by Muris (2002) and adapted into Turkish by Telef and Karaca (2012). Items included statements such as “How well do you succeed in passing all subjects?”. The academic self-efficacy subscale consists of 7 items, each rated on a 5-point Likert scale (1 = not at all, 5 = very well). Reliability and validity statistics for the present study are reported in Table 1.

Cronbach’s alpha coefficient was used to assess the internal consistency of the scales. According to Nunnally and Bernstein (1994), alpha values of 0.70 or higher indicate acceptable internal consistency. Another commonly used reliability indicator in structural equation modeling is composite reliability (CR). As recommended by Hair et al. (2014), CR values above 0.70 indicate adequate scale reliability. The average variance extracted (AVE) was also calculated to assess convergent validity, with values above 0.50 considered acceptable (Fornell & Larcker, 1981). As shown in Table 1, all scales exceeded the recommended thresholds for Cronbach’s alpha, CR, and AVE. These results indicate that the measurement instruments used in this study demonstrate adequate reliability and convergent validity.

### 3.3. Data Analysis

Structural equation modeling (SEM) was conducted using R version 4.5.1 (R Core Team, 2025) within the RStudio environment (version 2025.05.1+513) (RStudio Team, 2023). Key packages included *lavaan* (Rosseel, 2011), *semPlot* (Epskamp, 2012/2025), *semTools* (Jorgensen et al., 2022), *psych* (Revelle, 2024), *dplyr* (Wickham et al., 2023), and *apaTables* (Stanley, 2021). The hypothesized mediation model was estimated using maximum likelihood (ML) estimation. The indirect effect was specified within the SEM model and its statistical significance was evaluated based on the model-estimated standard errors.

Model fit was evaluated using multiple indices: χ^2^/df, CFI, TLI, RMSEA, and SRMR. Assumptions of normality and absence of multicollinearity were assessed using skewness, kurtosis, and variance inflation factor (VIF). To assess common method bias, Harman’s single-factor test and a single-factor confirmatory factor analysis (CFA) were performed.

### 3.4. Results

Confirmatory factor analyses (CFA) were conducted to examine the measurement properties of the latent constructs. The factorial structures of intolerance of uncertainty and academic self-efficacy were consistent with prior validation studies and demonstrated acceptable reliability and validity indicators. However, additional analyses were conducted for the academic resilience scale because the negative affect dimension did not demonstrate satisfactory model fit in the present sample. The comparison of CFA models is presented in Table 2.

As shown in Table 2, the original three-factor structure of the Academic Resilience Scale includes perseverance, adaptive help-seeking, and negative affect (Cassidy, 2016). A CFA including all three dimensions indicated inadequate model fit (CFI = 0.870, TLI = 0.837, RMSEA = 0.136, SRMR = 0.094). Inspection of standardized factor loadings and modification indices suggested localized areas of model misfit associated with the negative affect items. Therefore, an alternative two-factor model excluding the negative affect dimension was estimated. The revised model demonstrated substantially improved model fit (CFI = 0.983, TLI = 0.972, RMSEA = 0.091, SRMR = 0.029). Based on these results, academic resilience was operationalized using the perseverance and adaptive help-seeking dimensions in the subsequent structural analyses.

**Figure 1:**
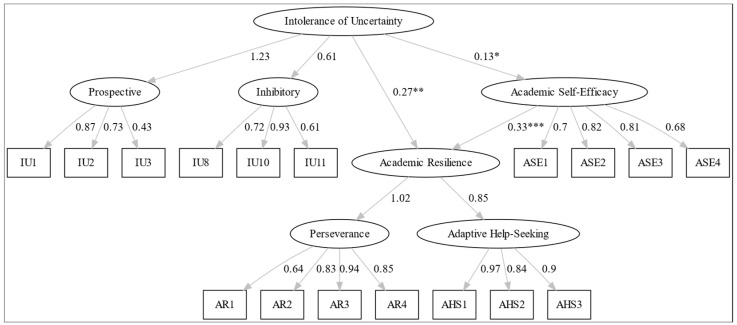
Structural equation model with standardized path coefficients. Note. Standardized beta coefficients indicate the strength of the relationships between variables. Asterisks indicate significance levels: * *p* < 0.05, ** *p* < 0.01, *** *p* < 0.001.

The hypothesized structural model (see Figure 1) demonstrated acceptable overall fit to the data (χ^2^/df = 3.76; CFI = 0.918; TLI = 0.900; RMSEA = 0.090; SRMR = 0.065). Although the RMSEA value slightly exceeds the commonly suggested 0.08 cutoff (Hu & Bentler, 1999), values in the 0.08–0.10 range are frequently interpreted as indicating mediocre but still acceptable model fit in SEM applications (MacCallum et al., 1996). Moreover, other fit indices (CFI, TLI, and SRMR) fall within recommended thresholds, suggesting that the model provides a reasonable representation of the observed data. RMSEA values are also known to be sensitive to model complexity and degrees of freedom, particularly in models with multiple latent constructs. The structural path estimates for the hypothesized model are presented in Table 3.

To examine whether the hypothesized model provided the most appropriate representation of the data, several theoretically plausible alternative models were tested. First, a first-order intolerance of uncertainty model was estimated in which all intolerance of uncertainty indicators loaded directly on a single first-order latent factor instead of a second-order structure. Second, an alternative mediation model was estimated in which academic self-efficacy predicted intolerance of uncertainty, which in turn predicted academic resilience. Third, a single-factor model was tested in which all observed indicators loaded onto a single latent construct to assess the potential influence of common method bias. Finally, a full mediation model was estimated by removing the direct path from intolerance of uncertainty to academic resilience. However, this model failed to converge and produced inadmissible parameter estimates. Therefore, it was not retained for interpretation. The comparison of structural models is presented in Table 4.

In addition to model fit indices, VIF values indicating no multicollinearity were 1.10 for intolerance of uncertainty and 1.06 for academic self-efficacy, indicating no multicollinearity (Hair et al., 2014). To evaluate the potential influence of common method bias, two complementary procedures were conducted. First, Harman’s single-factor test was performed using exploratory factor analysis. The results indicated that the first factor accounted for 33% of the total variance, which is below the commonly used threshold of 50%. Second, a single-factor confirmatory factor analysis (CFA) model was estimated in which all observed indicators were constrained to load onto a single latent construct. The model demonstrated a poor fit to the data (χ^2^ = 1740.12, df = 119, CFI = 0.569, TLI = 0.507, RMSEA = 0.200, SRMR = 0.168).

As shown in Figure 1, Hypothesis 1 (intolerance of uncertainty negatively predicts academic self-efficacy) was not supported. Although intolerance of uncertainty significantly predicted academic self-efficacy (β = 0.130, *p* < 0.05), the effect was positive rather than negative, contrary to expectations. Possible explanations for this unexpected finding are presented in the discussion section. The results support Hypothesis 2 (academic self-efficacy positively predicts academic resilience). Academic self-efficacy was found to be a significant positive predictor of academic resilience (β = 0.334, *p* < 0.001), indicating that individuals with higher self-efficacy are more likely to demonstrate resilience in the face of academic challenges.

Mediation analysis revealed that academic self-efficacy plays a mediating role in the relationship between intolerance of uncertainty and academic resilience; however, the direction of the effects contradicted the original hypothesis. The indirect effect was statistically significant (β = 0.044, *p* < 0.05), and the total effect was calculated as β = 0.311, *p* = 0.001. Although statistically significant, the magnitude of the indirect effect was small, indicating a limited mediating role of academic self-efficacy. Thus Hypothesis 3 (Academic self-efficacy mediates the relationship between intolerance of uncertainty and academic resilience) was partially supported.

Overall, the results suggest that academic self-efficacy is a significant positive predictor of academic resilience. Furthermore, intolerance of uncertainty influences self-efficacy, which thereby partially mediates its relationship with academic resilience.

## 4. Discussion

This study aimed to examine the relationships among vocational high school students’ intolerance of uncertainty, academic self-efficacy and academic resilience. The findings indicate that intolerance of uncertainty positively influences academic self-efficacy, which enhances academic resilience. Mediation analysis supports the indirect effect of intolerance of uncertainty on academic resilience through academic self-efficacy, indicating that academic self-efficacy partially mediates this relationship. The overall model demonstrated acceptable fit according to several indices; however, the RMSEA value (0.090) indicates only moderate level of model fit. Previous methodological literature suggests that RMSEA values between 0.08 and 0.10 may still represent mediocre but acceptable fit (Browne & Cudeck, 1993), and simulation studies have shown that cutoff values should not be interpreted as universal decision rules because model fit may vary depending on sample size, degrees of freedom, and model complexity (F. Chen et al., 2008). Nevertheless, the moderate RMSEA value suggests that the proposed model may not fully capture the complexity of the relationships among intolerance of uncertainty, academic self-efficacy, and academic resilience. This may reflect omitted variables or additional mechanisms not included in the present model. Future research should therefore consider incorporating other theoretically relevant constructs—such as social support, personality traits, or coping strategies—to improve the explanatory power and overall model fit. The findings demonstrate that academic self-efficacy has a positive effect on academic resilience. Academic self-efficacy is a significant predictor of academic resilience (Sabouripour et al., 2021) and students with high academic self-efficacy persist in the face of challenging situations and are better able to cope with difficulties. The findings support the conclusion that academic self-efficacy can enhance academic resilience (Cassidy, 2015). The present study extends the literature (Shao & Kang, 2022; Victor-Aigboidion et al., 2020; Uygur et al., 2023) by emphasizing the relationship between uncertainty intolerance and academic resilience in tourism and hospitality.

Contrary to the hypothesis intolerance of uncertainty was positively related to academic self-efficacy in this study. Although the literature has reported that intolerance of uncertainty is negatively related to academic self-efficacy, this relationship requires further exploration. Intolerance of uncertainty reflects a dispositional tendency to perceive ambiguity as threatening; however, its two dimensions—prospective and inhibitory—may operate differently in academic contexts. While inhibitory intolerance is linked to avoidance and withdrawal, prospective intolerance involves active information-seeking and cognitive engagement aimed at reducing ambiguity. From a transactional stress perspective (Lazarus & Folkman, 1984), intolerance of uncertainty primarily influences primary appraisal by increasing threat perception. However, secondary appraisal processes may activate compensatory coping strategies aimed at regaining control. In situations characterized by repeated exposure to uncertainty, individuals high in intolerance of uncertainty may engage in vigilant and effortful coping behaviors. According to uncertainty reduction theory, such individuals actively seek information and exert effort to reduce ambiguity (He et al., 2021). Over time, repeated engagement with uncertain academic demands may generate mastery experiences, which constitute a central source of self-efficacy beliefs (Bandura, 1997). Thus, rather than uniformly undermining academic self-efficacy, intolerance of uncertainty may, under certain conditions, mobilize proactive coping that strengthens competence beliefs. Intolerance of uncertainty results in stress and anxiety, while personality traits, cognitive flexibility, active coping styles, and social relationships and support are stated to play a role in how individuals deal with this condition (Carver & Connor-Smith, 2010; DeLongis & Holtzman, 2005; İnözü et al., 2023). These factors mitigate stress and anxiety levels stemming from intolerance of uncertainty and are pivotal in enhancing self-efficacy and academic resilience (Tang, 2019; Romano et al., 2021). Individuals with high levels of intolerance of uncertainty tend to adopt vigilant coping strategies that involve monitoring and evaluating negative thoughts (Grenier et al., 2005; Wu et al., 2013).

Social support for young adults has more powerful effect on resilience level compared to older adults (Gooding et al., 2012; MacLeod et al., 2016). With increasing uncertainty, individuals tend to develop greater trust in God, heroes, and political leaders in Turkish culture (Sargut, 2001). Moreover, Turkey’s collectivist culture leads to an increase in solidarity in situations where there is a general intolerance of uncertainty (Sümer et al., 2021). Social relationships and solidarity enhance individuals’ belief in their own abilities—in other words, their self-efficacy (Azpiazu et al., 2024; Kleppang et al., 2023). In particular, with regard to academic self-efficacy, it may be argued that solidarity among adolescents allows intolerance of uncertainty to exert a reinforcing rather than a detrimental effect on their academic self-efficacy. The frequent changes in Turkey’s education system—in which a policy implemented in one year may be no longer be valid the next—increase uncertainty. Adolescents may be exposed to these uncertainties as early as primary education and may, as a result, become accustomed to dealing with them and develop active coping strategies. Students in Turkey, who frequently experience uncertainty related to the education system, may exert greater effort to overcome such situations and thereby fostering academic self-efficacy. Taken together, these findings challenge the dominant deficit-based assumption that intolerance of uncertainty uniformly undermines adaptive academic beliefs. Instead, the results suggest that in collectivist and vocational educational contexts, intolerance of uncertainty may activate effortful coping processes that ultimately strengthen academic self-efficacy and, indirectly, academic resilience. Therefore, rather than simply reducing uncertainty, educational environments may also benefit from supporting stu-dents in developing adaptive coping resources—such as academic self-efficacy—that enable them to manage uncertain academic situations effectively.

Based on the study’s results, academic self-efficacy mediated the relationship between intolerance of uncertainty and academic resilience. Although the indirect effect of academic self-efficacy was statistically significant, the magnitude of this effect was relatively small. This suggests that academic self-efficacy plays a limited mediating role in the relationship between intolerance of uncertainty and academic resilience. Nevertheless, small indirect effects are frequently observed in mediation analyses of complex psychological processes where multiple variables jointly influence outcomes (Preacher & Hayes, 2008). Therefore, the present findings indicate that academic self-efficacy represents one possible mechanism through which intolerance of uncertainty may influence students’ academic resilience, while other psychological or contextual factors may also contribute to this relationship.

Vocational high school students with high intolerance of uncertainty are likely to adopt various coping strategies to manage such uncertainty. Their academic self-efficacy improves, and their academic resilience is partially strengthened by enhanced self-confidence. Vocational high school students who take both theoretical and practical courses (cooperative education) tend to feel more confident in their vocational abilities than other high school student.

Internship students are usually assumed to work autonomously, control themselves, and get support when necessary (Mikkonen et al., 2017; Pylväs et al., 2022). These conditions may strengthen their self-regulated learning skills. Self-regulated learning is one of the main constructs related to academic resilience (Cassidy, 2016). When vocational high school students engage in internship placements during specific periods of their educational program, their self-regulated learning, a critical predictor of academic resilience, is enhanced as well as their academic self-efficacy (Cassidy, 2016).

Given that production and consumption in the tourism sector occur concurrently, necessitating direct contact between guests and vocational high school students, these students are compelled to work autonomously and to manage their self-regulated learning. They boost practical learning and encourage student participation in processes that are beneficial for the workplace and the students themselves (Jossberger et al., 2010). In other words, students who actively participate in workplace activities and take on responsibilities tend to show increased academic resilience (Schmid & Haukedal, 2022). Their practical experience appears to sustain their motivation to address academic adversities, even when they experience elevated levels of intolerance of uncertainty, as workplace-based learning environments may enhance students’ engagement, confidence, and adaptive coping capacities (Hong et al., 2021; Weldon & Ngo, 2019). The findings of the present study indicate that in vocational education contexts, practical experience and workplace-based learning may help students transform uncertainty into challenge-oriented coping, thereby sustaining their academic motivation and resilience. Successful performance accompanied by positive feedback tends to heighten self-efficacy and subsequently promotes even greater achievement (Raelin et al., 2011; Brown et al., 2016). Prior research suggests that academic self-efficacy is more strongly associated with academic achievement than anxiety-related emotional responses (Pintrich & De Groot, 1990). Even if vocational high school students have high intolerance of uncertainty, they may still have high academic self-efficacy and perceive demanding situations as challenges, not threats (Chemers et al., 2001; Mega et al., 2014). Thus, when students perceive stressful events as threats, they have academic self-efficacy to overcome threats and begin to endure academic resilience (Hancock et al., 2015).

Another significant result of the study concerns the measurement structure of academic resilience. The confirmatory factor analysis indicated that the negative affect dimension of the Academic Resilience Scale did not demonstrate a satisfactory model fit in the present sample. While this decision improved the overall measurement validity, it may also have theoretical implications for how academic resilience is interpreted in this study. Specifically, the remaining dimensions primarily capture behavioral persistence and adaptive coping strategies in response to academic difficulties, whereas the emotional component of resilience is less represented. Consequently, academic resilience in the present study should be interpreted mainly in terms of students’ strategic and behavioral responses to academic challenges rather than their emotional regulation processes. Future research may further investigate the emotional dimension of academic resilience and examine whether this component operates differently across educational contexts or cultural settings.

The total academic resilience score was calculated based on two dimensions. Perseverance is a crucial sub construct of academic resilience and is strongly related to academic achievement and class attendance (A. Duckworth & Gross, 2014; Thorsen et al., 2021). Students with high intolerance of uncertainty appraise situations as more stressful than those with low intolerance of uncertainty. The perseverance dimension requires students to study harder to achieve goals and to avoid negative responses. Students who have high academic self-efficacy can reduce anxiety and avoid negative responses (Ramadhana et al., 2021).

The study recommends several points to the supervisors at the work and school teachers. Contrary to the literature, this study found that intolerance of uncertainty is positively related to academic self-efficacy, and subsequently to academic resilience. Although the level of intolerance of uncertainty has been increasing, personnel resources and contextual factors have major roles in fostering self-efficacy and then resilience (Schwarze & Wosnitza, 2018). Thus, teachers and supervisors should set realistic goals and give students responsibility, work autonomy and support both in the workplace and at the school. Moreover, supervisors/managers or teachers provide fruitful learning environments for internships that foster strong self-regulated learning skills which lead to academic self-efficacy and then academic resilience. The secure attachment style oriented psycho-educational program for adolescents in Turkey was implemented and has become effective in reducing the level of intolerance of uncertainty (Yıldız & Iskender, 2019). The psycho-educational program oriented toward a secure attachment style program should be implemented. Policy-makers should promote interventions to increase teachers’ wellbeing to give support to their students (Romano et al., 2021). In Turkey, where collectivist values such as solidarity, interdependence, and shared responsibility are deeply embedded, uncertainty may be mitigated through strong social support networks, peer collaboration, and family involvement. These collectivist mechanisms can enhance students’ sense of control and belief in their capacity to overcome adversity. From an equity and inclusion perspective, vocational students appear to actively engage with uncertainty by leveraging these cultural and social resources. Educational policies that recognize and reinforce such culturally rooted coping strategies can play a pivotal role in fostering equity, enhancing support for vocational learners, and ultimately reducing dropout rates.

This research has some limitations. First, this study uses a self-report approach to measure intolerance of uncertainty, academic self-efficacy and academic resilience among vocational high school students which may result in response bias. Future studies may consider collecting data at multiple time points or incorporating information from diverse sources to avoid this problem. Second, this study was conducted among vocational high school students in Istanbul. The findings cannot be applied to all high school students. Future scholars can examine similar topics across different types of high schools. Third, this study was conducted in Turkey, which has high level of collectivism. The results should therefore be considered, taking this into account. Future studies should be conducted in different cultures. Fourth, this study focuses only on the relationships among intolerance of uncertainty, academic self-efficacy and academic resilience. Personality related variables fall outside the focus of the study. Personality characteristics that buffer the adverse effects of stress and enhance individuals’ adaptation capacity are related with greater levels of resilience (Oshio et al., 2018). Well-being is another component that affects perception of threat and competing strategies. Social support and family support are important factors in coping with stressful events. Future studies should examine personality characteristics, well-being, social support and family support as moderators in these relationships. Finally, the sample characteristics should be considered when interpreting the findings. Although the study included 387 students, the response rate was relatively low, and participation was voluntary. Data collection took place in a school setting and required informed consent procedures, which may have reduced participation by some students. In addition, participation depended on students who were present in class during the data collection period and who were willing to complete the survey. In school-based surveys, female students are more likely to show slightly higher participation and classroom attendance, which may have contributed to the gender distribution observed in the sample. Female students tend to have higher levels of self-discipline and academic engagement than male students which may make them more willing to participate in academic activities and surveys (A. L. Duckworth & Seligman, 2005).

Furthermore, a large proportion of participants were final-year students, reflecting the accessibility of senior classes during the data collection period. These characteristics should be considered when interpreting the findings, and future studies should aim to include more balanced samples across genders and grade levels.

## 5. Conclusions

This study investigated the relationships among intolerance of uncertainty, academic self-efficacy, and academic resilience in vocational high school students within a collectivist cultural context. Contrary to expectations, the findings revealed a positive relationship between intolerance of uncertainty and academic self-efficacy, academic self-efficacy, in turn, significantly predicted academic resilience. Academic self-efficacy partially mediated the relationship between intolerance of uncertainty and academic resilience. The study suggests that students may develop adaptive coping mechanisms and self-confidence even under high levels of uncertainty—especially in environments enriched with social support and work-based learning opportunities. These findings contribute to the literature on the development of resilience in educationally disadvantaged groups and underscore the importance of self-regulated learning and cultural coping resources. However, the study’s reliance on self-report measures, its one-city sample, and the absence of measures of personality traits and support variables limit generalizability. Future research should explore these relationships across diverse school types, cultural contexts, personality variables and incorporate longitudinal and multi-source data to enhance robustness.

## Figures and Tables

**Table 1:** Reliability and Validity Estimates for Study Variables.

Variable	Cronbach’s α	Composite Reliability (CR)	Average Variance Extracted (AVE)
Intolerance of Uncertainty	0.82	0.86	0.55
Prospective Intolerance	0.70	0.75	0.52
Inhibitory Intolerance	0.80	0.80	0.58
Academic Self-Efficacy	0.83	0.83	0.55
Academic Resilience	0.93	0.94	0.72
Perseverance	0.88	0.89	0.66
Adaptive Help-Seeking	0.93	0.93	0.82

Note. Cronbach’s alpha (α) assesses internal consistency. Composite reliability (CR) and average variance extracted (AVE) evaluate the internal reliability and convergent validity of the constructs and their subdimensions.

**Table 2:** Comparison of Confirmatory Factor Analysis Models for Academic Resilience.

Model	χ^2^	df	χ^2^/df	CFI	TLI	RMSEA	SRMR
Three-factor model (perseverance, adaptive help-seeking, negative affect)	453.52	62	7.31	0.870	0.837	0.136	0.094
Two-factor model (perseverance, adaptive help-seeking)	49.88	13	3.84	0.983	0.972	0.091	0.029

Note. χ^2^ = chi-square statistic; df = degrees of freedom; CFI = Comparative Fit Index; TLI = Tucker–Lewis Index; RMSEA = Root Mean Square Error of Approximation; SRMR = Standardized Root Mean Square Residual; AIC = Akaike Information Criterion; BIC = Bayesian Information Criterion.

**Table 3:** Structural path estimates for the hypothesized model.

Path	B	SE	z	*p*	β
Intolerance of uncertainty → Academic self-efficacy	0.076	0.036	2.116	0.034	0.130
Academic self-efficacy → Academic resilience	0.402	0.075	5.395	<0.001	0.334
Intolerance of uncertainty → Academic resilience	0.187	0.058	3.213	0.001	0.267
Indirect effect	0.030	0.015	2.019	0.043	0.044
Total effect	0.217	0.065	3.351	0.001	0.311

**Table 4:** Model Specification and Comparison of Structural Models.

Model	Model Specification	χ^2^	df	χ^2^/df	CFI	TLI	RMSEA	SRMR
Hypothesized	IU → Academic Self-Efficacy → Academic Resilience + IU → Academic Resilience	421.63	112	3.76	0.918	0.900	0.090	0.065
First-order IU	IU measured as a single first-order latent factor	510.26	114	4.48	0.895	0.874	0.101	0.078
Alternative mediation	Academic Self-Efficacy → IU → Academic Resilience	451.02	113	3.99	0.910	0.892	0.094	0.102
Single-factor model	All observed indicators load onto one common latent factor	1740.12	119	14.62	0.569	0.507	0.200	0.168
Full mediation model	IU → Academic Self-Efficacy → Academic Resilience (no direct IU → Resilience path)	Did not converge	–	–	–	–	–	–

## Data Availability

The datasets generated during and analyzed during the current study are available from the corresponding author on request.
